# Supplementary material for: Radiomic Signatures Associated with CD8+ Tumour-Infiltrating Lymphocytes: A Systematic Review and Quality Assessment Study
Source: Cancers (Basel). 2022 Jul 27;14(15):3656. doi: 10.3390/cancers14153656 (PMC9367613; doi:10.3390/cancers14153656)
Supplement: Supplementary file 1 [file cancers-14-03656-s001.zip › SUPPLEMENTARY FIGURES (Ramlee, S et al).pdf]

Supplementary Materials

Radiomic Signatures Associated with CD8$^+$ Tumour-Infiltrating Lymphocytes: A Systematic Review and Quality Assessment Study

Syafiq Ramlee, David Hulse, Kinga Bernatowicz,
Raquel Pérez-López, Evis Sala, and Luigi Aloj

Figures

- **Figure S1**: QUADAS-2 and RQS assessments by the first reviewer.
- **Figure S2**: QUADAS-2 and RQS assessments by the second reviewer.

Tables (*refer to Excel sheet*)

- **Table S1**: PRISMA checklist.
- **Table S2**: Data extraction table for studies included in systematic review.
- **Table S3**: Study eligibility decision trail (for systematically-searched and manually-included articles) from the first reviewer.
- **Table S4**: Study eligibility decision trail (for systematically-searched and manually-included articles) from the second reviewer.
- **Table S5**: Description of technical radiomics terminology used in this systematic review.
- **Table S6**: List of relevant radiomics signatures from studies included in this systematic review.
- **Table S7**: Inter-rater agreement statistics for QUADAS-2 and RQS ratings.

QUADAS-2

Rater 1 (S.R.)

Study	Risk of bias: Patient selection	Risk of bias: Index test	Risk of bias: Reference standard	Risk of bias: Flow & timing	Applicability: Patient selection	Applicability: Index test	Applicability: Reference standard
Lopci (2016)	High	Low	Low	High	Low	Unclear	Low
Castello (2018)	High	Low	Low	High	Low	Unclear	Low
Sun (2018)	High	Low	Low	Unclear	Low	Low	Low
Chen (2019)	High	Low	Low	Low	Low	High	Low
Liao (2019)	High	Low	Low	Low	Low	High	Low
Zhang (2020)	High	Low	Low	Unclear	Unclear	High	Low
Jiang (2020)	High	Low	Low	Low	Low	Low	Low
Mazzaschi (2020)	High	Low	Low	High	Low	High	Low
Mitchell (2020)	High	High	Low	Low	Low	Unclear	Low
Katsoulakis (2020)	High	Low	Low	Unclear	Low	Low	Low
Hsu (2020)	High	High	Low	Unclear	Unclear	High	Low
Wen (2020)	High	Low	Low	Unclear	Low	High	Low
Zhang (2021)	High	Low	Low	Low	Low	Unclear	Low
Toulmonde (2020)	Unclear	Low	Low	Unclear	Low	Unclear	Low
Kim (2021)	Unclear	Low	Low	Unclear	High	Unclear	Low
Wang (2021)	High	Low	Low	Unclear	Low	High	High
Ligero (2021)	High	Low	Low	High	Low	Low	Low
Zhou (2021a)	High	Low	Low	Low	Low	Unclear	Low
Aoude (2021)	High	Low	Low	Unclear	Unclear	Unclear	Low
Arefan (2021)	High	Low	Low	Unclear	Unclear	High	Low
Li (2021)	High	Low	Low	Unclear	Low	High	Low
Bian (2021a)	High	Low	Low	Low	Low	High	Low
Jeon (2021)	High	Low	Low	Low	Low	High	Low
Chaddad (2021)	Unclear	Low	Low	Unclear	Low	High	Low
Bian (2021b)	High	Low	Low	Low	Low	High	Low
Min (2021)	High	Low	Low	Low	Low	High	Low
Zhou (2021b)	High	Low	Low	Low	Low	High	Low

High risk/concern
Low risk/concern
Unclear risk/concern

Radiomics Quality Score

Study	Imaging protocol quality	Multiple segmentation	Phantom study	Multi time-point imaging	Feature reduction/ robustness	Multivariable analysis	Biological correlates	Cut-off analyses	Discrimination statistics	Calibration statistics	Prospective study	Validation	'Gold standard' comparison	Potential clinical utility	Cost-effectiveness analysis	Open science	Overall RQS
Lopci (2016)	0	0	0	0	-3	1	1	1	0	0	0	-5	0	2	0	0	-3
Castello (2018)	0	0	0	0	-3	1	1	1	2	0	0	-5	2	2	0	0	1
Sun (2018)	1	1	0	0	3	1	1	1	2	0	0	5	2	2	0	0	19
Chen (2019)	1	1	0	0	3	1	1	1	2	1	0	2	2	2	0	0	17
Liao (2019)	1	1	0	0	3	1	1	1	2	0	0	2	2	2	0	0	16
Zhang (2020)	0	0	0	0	3	1	0	1	2	0	0	2	2	2	0	1	14
Jiang (2020)	1	1	0	0	3	1	1	1	2	2	0	5	2	2	0	1	22
Mazzaschi (2020)	0	0	0	0	3	1	1	1	2	0	0	2	2	2	0	0	14
Mitchell (2020)	0	1	0	0	-3	1	1	0	0	0	0	-5	2	2	0	0	-1
Katsoulakis (2020)	0	0	0	0	3	1	1	1	2	0	0	3	2	2	0	0	15
Hsu (2020)	0	0	0	0	3	1	1	1	1	0	0	2	2	2	0	0	13
Wen (2020)	1	1	0	0	3	1	0	0	2	0	0	2	2	2	0	0	14
Zhang (2021)	1	1	0	0	3	1	1	1	2	2	0	-5	2	2	0	0	11
Toulmonde (2020)	0	0	0	0	3	1	0	0	1	0	?	-5	0	2	0	0	2
Kim (2021)	0	0	0	0	3	1	1	1	2	0	?	-5	2	2	0	0	7
Wang (2021)	0	0	0	0	3	1	1	1	2	0	0	2	2	2	0	0	14
Ligero (2021)	1	1	0	0	3	1	1	1	2	2	0	3	2	2	0	0	19
Zhou (2021a)	0	0	0	1	3	1	1	1	2	0	0	-5	2	2	0	0	8
Aoude (2021)	0	0	0	0	-3	1	0	0	0	0	0	-5	2	2	0	0	-3
Arefan (2021)	1	0	0	0	3	1	0	1	1	0	0	2	0	2	0	0	11
Li (2021)	1	1	0	0	3	1	1	1	2	2	0	2	2	2	0	0	18
Bian (2021a)	1	1	0	0	3	1	0	1	2	2	0	2	2	2	0	0	17
Jeon (2021)	0	1	0	0	3	1	1	1	2	0	0	2	2	2	0	0	15
Chaddad (2021)	0	0	0	0	3	1	1	1	2	0	?	2	2	0	0	0	12
Bian (2021b)	1	1	0	0	3	1	0	1	2	2	0	2	2	2	0	0	17
Min (2021)	0	1	0	0	3	1	1	1	1	0	0	2	0	2	0	0	12
Zhou (2021b)	0	1	0	0	3	1	1	1	2	2	0	2	2	2	0	0	17
Average	0.41	0.52	0	0.04	2.11	1.00	0.74	0.85	1.63	0.56	0	0.22	1.70	1.93	0	0.07	11.78

Figure S1: QUADAS-2 and RQS assessments by the first reviewer.

QUADAS-2

Rater 2 (D.H.)

Study	Risk of bias: Patient selection	Risk of bias: Index test	Risk of bias: Reference standard	Risk of bias: Flow & timing	Applicability: Patient selection	Applicability: Index test	Applicability: Reference standard
Lopci (2016)	High	Low	Low	High	Low	Low	Low
Castello (2018)	High	Low	Low	Unclear	Low	Low	Low
Sun (2018)	High	Low	Low	Low	Low	High	Low
Chen (2019)	High	Low	Low	Low	Low	High	Low
Liao (2019)	High	Unclear	Low	Unclear	Unclear	High	Low
Zhang (2020)	High	Low	Low	Unclear	Low	Unclear	Low
Jiang (2020)	High	Low	Low	High	Low	High	Low
Mazzaschi (2020)	High	High	Low	Low	Low	Unclear	Low
Mitchell (2020)	High	Low	Low	Unclear	Low	Unclear	Low
Katsoulakis (2020)	High	Low	Low	Unclear	Low	High	Low
Hsu (2020)	High	Low	Low	Low	Low	Unclear	Low
Wen (2020)	High	Low	Low	Unclear	Unclear	Unclear	Low
Zhang (2021)	High	Low	Low	Unclear	Unclear	High	Low
Toulmonde (2020)	High	Low	Low	Low	Low	High	Low
Kim (2021)	High	Low	Low	Low	Low	High	Low
Wang (2021)	High	Low	Low	Low	Low	High	Low
Ligero (2021)	High	Low	Low	Unclear	Low	High	Low
Zhou (2021a)	High	Low	Low	Unclear	Low	High	Low
Aoude (2021)	High	Low	Low	High	Low	Unclear	Low
Arefan (2021)	High	Low	Low	Unclear	Low	High	Low
Li (2021)	High	Low	Low	Unclear	Low	High	Low
Bian (2021a)	High	Low	Low	Low	Low	High	Low
Jeon (2021)	High	Low	Low	Low	Low	High	Low
Chaddad (2021)	High	Low	Low	High	Low	Unclear	Low
Bian (2021b)	High	Low	Low	Low	Low	Unclear	Low
Min (2021)	High	Low	Low	Unclear	Low	High	Low
Zhou (2021b)	High	Low	Low	Unclear	Low	High	Low

High risk/concern
Low risk/concern
Unclear risk/concern

Radiomics Quality Score

Study	Imaging protocol quality	Multiple segmentation	Phantom study	Multi time-point imaging	Feature reduction/ robustness	Multivariable analysis	Biological correlates	Cut-off analyses	Discrimination statistics	Calibration statistics	Prospective study	Validation	'Gold standard' comparison	Potential clinical utility	Cost-effectiveness analysis	Open science	Overall RQS
Lopci (2016)	0	0	0	0	-3	1	1	1	1	0	0	-5	2	2	0	0	0
Castello (2018)	0	0	0	0	-3	1	1	1	2	0	0	-5	2	2	0	0	1
Sun (2018)	1	1	0	0	3	1	1	1	2	1	0	4	2	2	0	0	19
Chen (2019)	1	1	0	0	3	1	1	1	2	0	0	2	2	2	0	0	16
Liao (2019)	0	0	0	0	3	1	1	1	1	0	0	2	2	2	0	0	13
Zhang (2020)	0	0	0	0	3	1	0	1	2	0	0	3	2	2	0	1	15
Jiang (2020)	1	1	0	0	3	1	1	1	2	2	0	5	2	2	0	1	22
Mazzaschi (2020)	0	0	0	0	3	1	1	1	2	0	0	2	2	2	0	0	14
Mitchell (2020)	0	0	0	0	-3	1	1	0	1	0	0	-5	0	2	0	0	-3
Katsoulakis (2020)	1	1	0	0	3	1	1	0	2	0	0	2	2	2	0	0	15
Hsu (2020)	0	0	0	0	3	1	0	1	2	0	0	2	2	2	0	0	13
Wen (2020)	1	1	0	0	3	1	0	0	1	0	0	2	2	2	0	0	13
Zhang (2021)	0	0	0	0	3	1	0	1	1	0	0	-5	0	2	0	0	3
Toulmonde (2020)	2	0	0	0	3	1	0	0	1	1	0	-5	0	2	0	0	5
Kim (2021)	0	0	0	0	3	1	1	1	2	0	0	-5	2	2	0	0	7
Wang (2021)	0	1	0	0	3	1	1	1	2	0	0	2	2	2	0	0	15
Ligero (2021)	1	1	0	0	3	1	1	1	2	1	0	3	2	2	0	0	18
Zhou (2021a)	1	1	0	0	3	1	1	1	2	2	0	-5	2	2	0	0	11
Aoude (2021)	0	0	0	0	-3	1	1	1	2	0	0	-5	2	2	0	0	1
Arefan (2021)	0	1	0	0	3	1	1	1	1	0	0	2	0	2	0	0	12
Li (2021)	0	0	0	0	3	1	1	1	2	0	0	2	2	2	0	0	14
Bian (2021a)	1	1	0	0	3	1	1	1	2	2	0	2	2	2	0	0	18
Jeon (2021)	1	1	0	0	3	1	1	1	2	0	0	2	2	2	0	0	16
Chaddad (2021)	0	0	0	0	3	1	1	1	2	0	0	3	2	0	0	1	14
Bian (2021b)	1	1	0	0	3	1	1	1	2	2	0	2	2	2	0	0	18
Min (2021)	0	0	0	0	3	1	1	1	2	0	0	2	2	0	0	0	12
Zhou (2021b)	1	1	0	0	3	1	1	1	2	2	0	2	2	2	0	0	18
Average	0.48	0.48	0	0	2.11	1.00	0.81	0.85	1.74	0.48	0	0.22	1.70	1.85	0	0.11	11.85

Figure S2: QUADAS-2 and RQS assessments by the second reviewer.
